# In silico functional prediction of hypothetical proteins from the core genome of *Corynebacterium pseudotuberculosis* biovar *ovis*

**DOI:** 10.7717/peerj.9643

**Published:** 2020-08-26

**Authors:** Carlos Leonardo Araújo, Iago Blanco, Luciana Souza, Sandeep Tiwari, Lino César Pereira, Preetam Ghosh, Vasco Azevedo, Artur Silva, Adriana Folador

**Affiliations:** 1 Laboratory of Genomics and Bioinformatics, Center of Genomics and Systems Biology, Institute of Biological Sciences, Federal University of Pará, Belém, Pará, Brazil; 2 Institute of Biological Sciences, Federal University of Minas Gerais, Belo Horizonte, Minas Gerais, Brazil; 3 Department of Computer Science, Virginia Commonwealth University, Richmond, VA, USA

**Keywords:** Hypothetical proteins, Functional annotation, *Corynebacterium pseudotuberculosis*, Caseous lymphadenitis

## Abstract

*Corynebacterium pseudotuberculosis* is a pathogen of veterinary relevance diseases, being divided into two biovars: *equi* and *ovis*; causing ulcerative lymphangitis and caseous lymphadenitis, respectively. The isolation and sequencing of *C. pseudotuberculosis* biovar *ovis* strains in the Northern and Northeastern regions of Brazil exhibited the emergence of this pathogen, which causes economic losses to small ruminant producers, and condemnation of carcasses and skins of animals. Through the pan-genomic approach, it is possible to determine and analyze genes that are shared by all strains of a species—the core genome. However, many of these genes do not have any predicted function, being characterized as hypothetical proteins (HP). In this study, we considered 32 *C. pseudotuberculosis* biovar *ovis* genomes for the pan-genomic analysis, where were identified 172 HP present in a core genome composed by 1255 genes. We are able to functionally annotate 80 sequences previously characterized as HP through the identification of structural features as conserved domains and families. Furthermore, we analyzed the physicochemical properties, subcellular localization and molecular function. Additionally, through RNA-seq data, we investigated the differential gene expression of the annotated HP. Genes inserted in pathogenicity islands had their virulence potential evaluated. Also, we have analyzed the existence of functional associations for their products based on protein–protein interaction networks, and perform the structural prediction of three targets. Due to the integration of different strategies, this study can underlie deeper in vitro researches in the characterization of these HP and the search for new solutions for combat this pathogen.

## Introduction

*Corynebacterium pseudotuberculosis* is a Gram-positive, facultative intracellular, non-capsulated, non-sporulating and non-motile organism. It is a member of the CMNR group (*Corynebacterium, Mycobacterium, Nocardia* and *Rhodococcus*), which is composed by bacteria that share common characteristics, such as: cell wall organization—mainly composed by peptidoglycan, arabinogalactan and mycolic acids—and a G+C content of its genomes varying from 47% to 74% (Dorella et al., 2006).

According to the ability on nitrate reduction, this species can be classified in two biovars, responsible for causing different clinical conditions. The biovar *equi* is composed by nitrate reductase positive bacteria, that are able to transmit ulcerative lymphangitis, which attacks mainly horses and buffaloes, and the biovar *ovis*, characterized as nitrate reductase negative isolates, can transmit caseous lymphadenitis (CLA). This disease is characterized by the formation of abscesses in lymph nodes and other internal organs of small ruminants, as goats and sheep, since these species generally are unable to perform nitrate reduction (Williamson, 2001; Dorella et al., 2006).

Caseous lymphadenitis entails harmful effects in the agribusiness globally, affecting the production of wool, meat and milk, besides diminishing the reproductive efficiency and condemning carcasses and skins of animals (Fontaine & Baird, 2008). In the Northeastern region of Brazil, *C. pseudotuberculosis* is responsible for infecting up to 37% of goats in animal farms, reinforcing the need for researches concerning this pathogen (Alves et al., 2018). Thus, it is necessary to perform in-depth research for the better comprehension of its biology, especially regarding to pathogenicity mechanisms—once the therapeutic methods current available are not completely effective, requiring prophylactic measures in order to combat proliferation and permanence of the pathogen into the hosts; however, those measures demand highly laborious processes (Lee, Hefta & Brennan, 1992).

Some bacterial genes can act as virulence factors and are related to pathogenesis, as the secreted exotoxin phospholipase D (PLD), which can be found in pathogenicity islands of the bacterial genome—specific *loci* containing genes related to pathogenicity. It is reported that PLD can have its expression affected by environmental factors such as temperature, pH and osmolarity (McKean, Davies & Moore, 2007; Ruiz et al., 2011). Also, the resistance to various environmental stresses can be associated to pathogenic bacteria survival and persistence in the host (Pinto et al., 2014). Considering the role of genes related to pathogenicity in the genome of *C. pseudotuberculosis*, the prospection and characterization of genetic targets related to the disease shows vital importance.

In the past years it has been noted the significant growth of genome projects in public databases—allowing the exponential development of studies related to comparative genomics, especially using pan-genomics. This approach is characterized by the classification of genes in three groups: the core genome, which is composed by genes shared by all strains; the accessory genome, which is composed by genes absent in some strains; and the strain-specific genes (Tettelin et al., 2005). Given this, the analysis of genes belonging to the core genome is particularly important, once the presence of orthologues shared by all strains of a biovar can reveal interesting objects of study in the search for new therapeutic or vaccine targets for CLA (Araújo et al., 2019).

Followed by the emergence of isolation and sequencing of new *C. pseudotuberculosis* biovar *ovis* genomes, especially in the Northern and Northeastern regions of Brazil (Alves et al., 2016; Muge et al., 2016), the development of strategies in order to comprehend and elucidate its genome features became bigger and faster, helping to understand biological processes, gene repertoire, gene regulation and PPI (Nourani, Khunjush & Durmus, 2015). Despite this, several genes present in the genome of this pathogen still have unknown functions, being characterized as hypothetical proteins (HP).

Hypothetical proteins are predicted by homologous sequences, but lack biological and chemical evidence. In *Mycobacterium tuberculosis*, as example, around 25% of the genes have no confirmed function, which can be involved in intracellular survival and progression of the disease (Yang, Zeng & Tsui, 2019). Currently, through bioinformatic tools, it is possible to predict the role of these proteins in bacteria and functionally annotate them by means of identification of conserved motifs and domains, analysis of protein interactions, gene expression data, and elucidation of physicochemical parameters.

Computational structural and functional analysis of HP have been proposed to a variety of bacteria—*Treponema pallidum* subspecies pallidum (Houston et al., 2018), *Prochlorococcus marinus* (Sai Arun et al., 2020), *Staphylococcus aureus* (Mohan & Venugopal, 2012), *Bacillus anthracis* (Anderson, 2009); parasites—*Leishmania donovani* (Ravooru et al., 2014), *Plasmodium falciparum* (Oladele, Sadiku & Bewaji, 2011) and viruses such as Human Adenovirus (Naveed et al., 2017). These data can provide better understanding of the pathogen’s physiology, its role during infection and survival in the host, besides assisting in the comprehension of biochemical pathways and their function in *C. pseudotuberculosis* (Lubec et al., 2005). Thus, the main purpose of this work is assigning function, through the integration of different in silico strategies, to sequences annotated as HP present in the core genome of *C. pseudotuberculosis* biovar *ovis*.

## Materials and Methods

**Figure 1:**
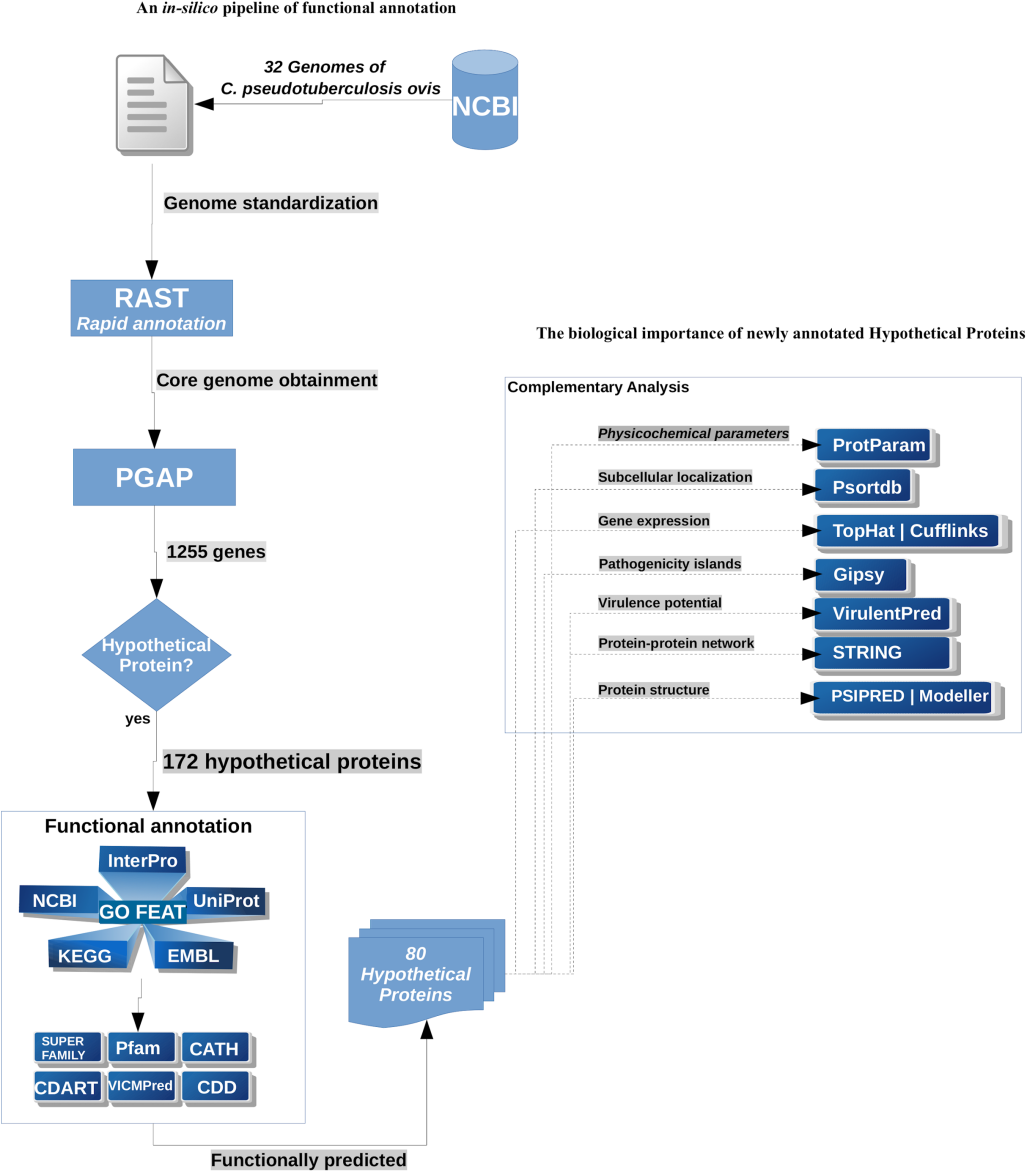
Workflow used for the annotation of the HP from *C*. *pseudotuberculosis* biovar *ovis*.

The workflow used for the prediction of the HP present in the core genome of *C. pseudotuberculosis* biovar *ovis* is described in Fig. 1. Briefly, we adopted two different strategies: (1) the pipeline of functional annotation itself—which included the search in several databases—and (2) the analysis of the newly annotated products to investigate its biological importance, through the use of different in silico approaches.

### Functional annotation

#### Retrieval of genome data

In this work, 32 complete genomes from *C. pseudotuberculosis* biovar *ovis* were used. All the genomes were retrieved from NCBI Genomes database (https://www.ncbi.nlm.nih.gov/genome/), and standardized by the online platform RAST (Overbeek et al., 2014). The main features of the strains used in our study are listed in Table 1.

**Table 1:** List of genomes used for pan-genomic analysis in *C. pseudotuberculosis* biovar *ovis*.

Strain	Size (Mb)	Genes	Isolation country	Host	NCBI Access number
1002B	2.3351	2138	Brazil	Goat	CP012837.1
12C	2.3374	2143	Brazil	Sheep	CP011474.1
226	2.3378	2143	USA	Goat	CP010889.1
267	2.3376	2141	USA	Llama	CP003407.1
29156	2.3386	2147	Israel	Bovine	CP010795.1
3/99-5	2.3379	2144	UK	Sheep	CP003152.1
42/02-A	2.3376	2143	Australia	Sheep	CP003062.1
C231	2.3282	2133	Brazil	Sheep	CP001829.1
E55	2.3353	2140	Egypt	Sheep	CP014341.1
E56	2.3357	2141	Egypt	Sheep	CP013699.1
FRC41	2.3379	2144	France	Human	CP002097.1
Ft_2193/67	2.3383	2145	Norway	Goat	CP008924.1
I19	2.3375	2143	Israel	Bovine	CP002251.1
MEX1	2.3370	2145	Mexico	Goat	CP017711.1
MEX25	2.3375	2143	Mexico	Sheep	CP013697.1
MEX29	2.3378	2145	Mexico	Sheep	CP016826.1
MEX9	2.3375	2146	Mexico	Goat	CP014543.1
MIC6	2.3371	2143	Brazil	Sheep	CP019769.1
N1	2.3378	2142	Guinea	Sheep	CP013146.1
P54B96	2.3376	2143	South Africa	Wildebeest	CP003385.1
PA01	2.3379	2141	Brazil	Sheep	CP013327.1
PA02	2.3284	2130	Brazil	Goat	CP015309.1
PA04	2.3380	2142	Brazil	Sheep	CP019587.1
PA05	2.4354	2247	Brazil	Sheep	CP019159.1
PA06	2.3204	2131	Brazil	Sheep	CP019289.1
PA07	2.3373	2146	Brazil	Sheep	CP024457.1
PA08	2.3377	2147	Brazil	Sheep	CP024602.1
PAT10	2.3353	2140	Argentina	Sheep	CP002924.1
PO22241	2.3375	2145	Portugal	Goat	CP013698.1
PO2695	2.3371	2145	Portugal	Goat	CP012695.1
T1	2.3372	2143	Brazil	Goat	CP015100.1
VD57	2.3371	2141	Brazil	Goat	CP009927.1

#### Obtainment of the core genome

After the standardization, we generated for each genome the .nuc. .pep and .function files, containing information of nucleotides, amino acids (AAs) and function of the coding sequences (CDS), respectively. These files were submitted to the software PGAP, which performs several functions, as analysis of functional gene clusters—classifying them onto core genome, accessory genome or strain-specific genes—besides analyzing the pan-genome profile, functional gene variation and species evolution (Zhao et al., 2012). For this analysis, we adopted the Gene Family (GF) method to the identification of orthologues genes, using a minimum score of blastall 40 (default parameter), 80% identity, 90% coverage and *e*-value of 0.00001. Subsequently, the CDS of the core genome identified as HP were extracted using the Artemis software (Rutherford et al., 2000).

#### Functional annotation of HP

At first, HP sequences were submitted to the online tool GO FEAT (Araujo et al., 2018) in order to search for homology in public databases, and integrated annotation data. Proteins were classified according to length, completeness, Gene Ontology (GO) terms, and the assignment of function was done through conservation of domains, motifs, families and superfamilies by identity with InterPro, UniProt, EMBL, KEGG and NCBI databases. Through a multi-fasta file of the 172 HP previous identified, we performed this preliminary GO based-annotation, using the BLASTp (*e*-value 0.001).

Subsequently, the sequences that presented hits on GO FEAT (GO terms) were then selected to be analyzed in another public databases for families, domains, motifs and function assignment, as Conserved Domain Database (Lu et al., 2020), SUPERFAMILY (Gough et al., 2001), Pfam (Finn et al., 2014), PROSITE (Sigrist et al., 2009), CATH (Sillitoe et al., 2015), CDART (Geer et al., 2002) and VICMPred (Saha & Raghava, 2006). In this step, we considered the default parameters of each database.

### Biological importance of the newly annotated products

#### Prediction of physicochemical parameters

The physicochemical characteristics of HP can provide insights to the elucidation of its products and further in vitro assays. ProtParam predicts the molecular weight (MW, given in Da), isoelectric point (IP), number of AAs (No of AA), instability index (II) and atomic composition of the targets. This tool also calculates other parameters, as the extinction coefficient (EC, given in M^−1^cm^−1^), useful in spectrophotometry experiments, aliphatic index (AI), related with thermostability and grand average of hydropathy (GRAVY), that indicates the interaction of proteins with water (Gasteiger et al., 2005).

#### Determination of the subcellular localization

We used PSORTdb 3.0 (Yu et al., 2010) to assign the subcellular localization of HP. This tool resorts the Support Vector Machine (SVM) approach, generating scores relative to each protein subcellular classifier based on the sequence of AAs, and evaluating the probabilities to determine a final location. PSORTdb was adopted because contains information of in vitro experiments and in silico predictions. We used an *e*-value cutoff of 0.001, through the algorithm BLASTp on the cPORTdb Blast Database.

#### Analysis of HP gene expression profile in different abiotical stress conditions

In order to enrich our findings, we decided to investigate if the predicted HP play role in the gene expression of *C. pseudotuberculosis* under conditions that simulate the phagolysosomal environment: acidic (pH 5), thermal (50 °C) and osmotic (2M) stresses. On this step, we analyzed the transcriptomic data of strain 1002, obtained by Pinto et al. (2014) and publically available at the NCBI SRA database (https://www.ncbi.nlm.nih.gov/sra?term=ERP004224).

We looked specifically for those targets that presented differential gene expression compared with the control condition, through the protocols of TopHat (Kim & Salzberg, 2011) and Cufflinks (Trapnell et al., 2012) software. At first, on TopHat we used the default parameters, besides the option *no-novel-juncs*, that mapped the reads of each condition and compared against the reference genome. Then, also applying the default parameter, the tool Cuffdiff, integrated on the Cufflinks pipeline, obtained the expression level values of Fragments Per Kilobase Of Exon Per Million Fragments Mapped (FPKM), allowing the identification of genes that have a significant differential expression, based on the Poisson distribution (cut-off values of *p*-value and *q*-value ≤ 0.05). For our analysis, we investigated the induced genes (fold change ≥ 2) in at least one of the three stress conditions.

#### Analysis of virulence potential of HP

Since some of the annotated products can possibly be involved in pathogenicity mechanisms, we evaluated its virulence potential through the online tool VirulentPred (Garg & Gupta, 2008), which is able to calculate a score of virulence potential, distinguishing molecules into virulent and non-virulent according to the threshold of 0.4, considered its default parameter.

#### Determination of presence of HP in pathogenicity islands in *C. pseudotuberculosis*

The software GIPSy (Soares et al., 2015) could perform analysis to determine the existence of genomic islands. Through comparison with the non-pathogenic genome (*C. glutamicum* ATCC13032) as reference, this tool is able to identify islands for pathogenicity, metabolism, resistance and symbiosis. After this analysis, it was evaluated the presence of target proteins of this study in these *loci* predicted by GIPSy.

#### PPI networks

The functionally annotated proteins were submitted to the STRING database (Szklarczyk et al., 2015). This database includes direct (physical) and indirect (functional) associations through a computational forecast. In our analysis, we considered only high-reliability interactions, with scores above 0.7 and maximum 10 interactions, to ensure the confidence of association. The interaction networks were generated based on the *C. pseudotuberculosis* E19 reference genome.

##### Determination and validation of protein structure

The secondary structure of the protein analyzed was predicted by the PSIPRED 4.0 (McGuffin, Bryson & Jones, 2000). Besides that, the environment of the PSIPRED server covers tools as GenTHREADER, used for fold recognition, based on the search for homology on Protein Data Bank (PDB) database and DomPred, for domain prediction (Buchan & Jones, 2019). For all these analyses we adopted the default parameters.

The Phyre2 web-based homology modelling server (Kelley et al., 2015) was used for the prediction of the proteins three-dimensional (3D) structure. The 3Drefine server (Bhattacharya et al., 2016) was used to refine the selected 3D structure model predicted by Phyre2 web serverand PROCHECK program—included on the SAVES 5.0 server pipeline (http://servicesn.mbi.ucla.edu/SAVES/)—was used to check and validate the stereochemical quality of 3D protein structures through the Ramachandran plot. The validation is based on the dihedral angles, which are used to specify the molecular conformation. The torsion angles ψ and φ determine the stability of the amino acid residues in the protein structure (Morris et al., 1992; Laskowski et al., 1993). Subsequently, models were visualized using UCSF Chimera 1.1.2 (Pettersen et al., 2004).

## Results

### Pan-genome profile

The results showed that the pan-genome of *C. pseudotuberculosis* biovar *ovis* contains 4,806 genes, where 1,255 are shared by all strains considered in this study (core genome); 1,516 compose the accessory genome; and 2,035 are strain-specific genes (Table S1). Among the components of the core genome, 172 genes were predicted as HP (13.7%). The amino acid sequences of these genes were obtained through the Artemis software and submitted to the annotation workflow for proper characterization of their functions.

### Physicochemical parameters and protein function

It is known that in silico analysis can reveal molecular importance, as some parameters are related to protein stability and function. Regarding the ProtParam results, the length of analyzed sequences ranged from 59 to 898 AAs, with a mean value of 227.3 AAs. The molecular weights ranged from 6,167.5 to 99,696.8 Da, with a mean value of 24,739.1 Da. The theoretical IPs ranged from 3.9 to 11.7, with a mean value of 6.5. This parameter relates to the point at which the amino acid does not tolerate liquid charge, not moving in an electric field of a direct current (Islam et al., 2015). The extinction coefficients at 280 nm ranged from 1,490 to 169,290, with a mean value of 28,615.1. The values for II ranged from 8.4 to 73, with a mean value of 37.1 and a cutoff of 40 to determine protein stability. Thus, we obtained 109 stable and 63 unstable proteins under normal conditions. Values for AIs ranged from 29 to 146.3, with a mean value of 94.5. This parameter is related to the thermostability of proteins, being directly proportional to the temperature range for which this molecule will be stable.

At last, GRAVY values ranged from −1.5 to 1.4, with a mean value of −0.025. Protein–water interactions occur better in low GRAVY (Da Costa et al., 2018). Hence, it was analyzed protein subcellular localizations, and observed that 75 belong to the cytoplasm, 78 in the cytoplasmic membrane, 2 in the cell wall and 17 are extracellular. Concerning to the role of these proteins, 92 were related to cellular processes, 73 to metabolism, 5 to virulence and 2 were classified as information molecules. The details of these data are shown on Table S2.

### Annotation of HP through integration of conserved structural data

**Figure 2:**
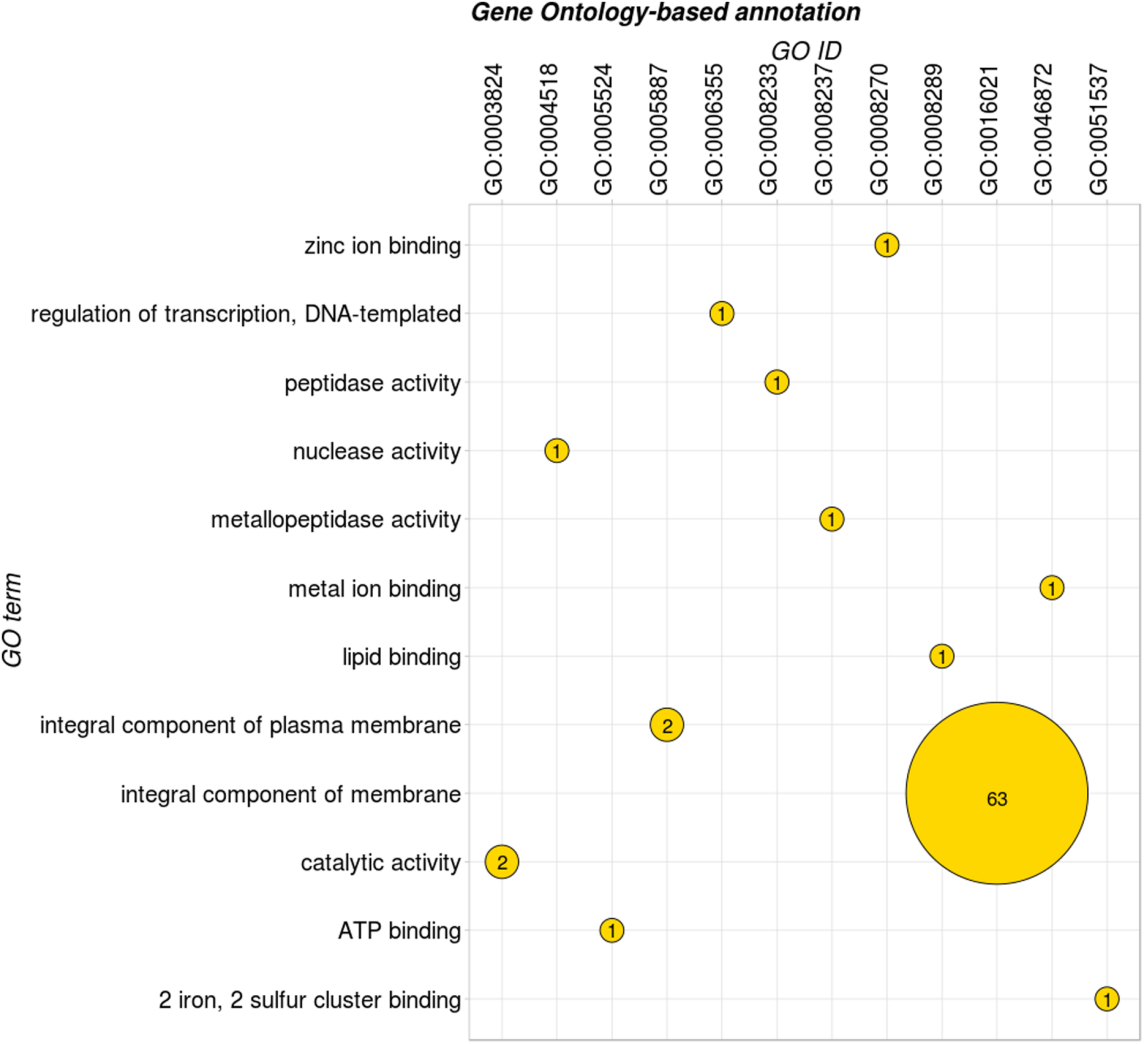
Main GO terms associated to the HP predicted in the GO FEAT platform.

Sequences were submitted to a previous annotation onto the GO FEAT platform. Among the 172 targets, 76 of them exhibited results on GO database (Fig. 2), represented by 12 GO terms, in three categories: molecular function, biological process and cell component. For each category, some GO subcategories were considered, as metabolic process, regulation of transcription, metal ion binding, catalytic activity, among others. The complete result of the preliminary annotation performed by GO feat can be consulted in Table S3.

Among the 76 GO-termed proteins, 11 were classified as molecular function, 63 as cell components and 2 as biological process related molecules. We must highlight that the great quantity of proteins classified as cell components may be due to the majority of the HP being considered as integral components of membrane. The cell membrane has an important role in physiology of Gram-positive bacteria as *C. pseudotuberculosis*, once it relates to functions as organism adaptation and survival in extreme environmental conditions (Lee, Hefta & Brennan, 1992).

The conservation of structural features could suggest function for some HP and its products for functional annotation. These structural features were found by integration of data available from online databases, considering conserved domains, families and superfamilies. These features can suggest important roles for the molecule, involved in cell process, metabolic activity or molecular affinity, for example. It is interesting to highlight that, even for some proteins that we could not annotate, it was possible to identify some structural features conserved in public databases. Thus, from the 172 HP, 80 could have assigned function through functional annotation, as listed in Table 2. However, the analysis through amino acid sequence can suggest significant information regarding to its functions, what can be ensured through auxiliary tools.

**Table 2:** List of annotated products for HP in the core genome of *C. pseudotuberculosis* biovar *ovis*.

Locus tag	UNIPROT ID	Product
Cp1002B_0016	A0A1L3KSD4	Cadherin repeat binding protein
Cp1002B_0046	D9QD92	SMAD/FHA superfamily protein
Cp1002B_0049	D9QD94	Multidrug efflux transporter AcrB
Cp1002B_0096	A0A0E3N3X9	Heavy-metal binding YbjQ-like protein
Cp1002B_0107	D9QDM3	PrsW peptidase
Cp1002B_0178	D9QDT9	Thioester transmembrane protein
Cp1002B_0182	D9QDU3	Permeases of the drug/metabolite transporter (DMT)
Cp1002B_0192	D9QE91	LPXTG cell wall anchor repeat
Cp1002B_0212	D9QDY6	Holin-X, superfamily III protein
Cp1002B_0235	D9QDZ4	Flp_Fap protein
Cp1002B_0263	D9QE21	ABC-transporter EcsB protein
Cp1002B_0335	D9QE89	LPXTG cell wall anchor repeat
Cp1002B_0341	D9QE95	Porin PorA family protein
Cp1002B_0349	D9QEA2	Cation efflux family protein
Cp1002B_0360	D9QEB2	Abhydrolase 9_N
Cp1002B_0362	D9QEB4	Cell envelope-related protein, Asp23 family
Cp1002B_0364	D9QEB6	Cell envelope-related protein, Asp23 family
Cp1002B_0418	D9QEG7	PGPW transmembrane protein
Cp1002B_0422	D9QEH2	Rv3446c secretion system type 7
Cp1002B_0436	D9QEI5	Holin-X protein, superfamily III
Cp1002B_0449	D9QEJ5	Permease aminoacid transporter
Cp1002B_0451	D9QEJ7	Immunity-68 protein
Cp1002B_0455	D9QEK1	AMIN Domain
Cp1002B_0472	D9QEL7	Restriction endonuclease HNHc
Cp1002B_0479	D9QEM3	HtaA Protein
Cp1002B_0510	D9QEQ3	Acyl-Coa carboxylase, subunity epsilon
Cp1002B_0531	D9QES3	Zincin-like metallopeptidase
Cp1002B_0565	D9QEV6	Transmembrane zincin-like metalloprotease
Cp1002B_0603	D9Q936	Tripsyin-like protease
Cp1002B_0654	D9QD03	HtaA protein
Cp1002B_0682	D9QCX7	Arabinan β-1,2-arabinofuranosyltransferase AftB
Cp1002B_0702	D9QCV7	Porin PorA channel-forming protein
Cp1002B_0719	D9QCU2	EamA-like transporter Family
Cp1002B_0792	D9QCW7	PLU-1-like protein
Cp1002B_0811	A0A1L6D2J8	Chemotaxis methyl-accepting receptor HlyB-like
Cp1002B_0820	D9QCJ2	LytR cell envelope-related transcriptional attenuator
Cp1002B_0871	D9QCE2	Putative threonine/serine exporte ThrE_2
Cp1002B_0894	D9QCC1	Restriction endonuclease-like superfamily
Cp1002B_0931	D9QC87	Thiamin pyrophosphokinase, catalytic domain
Cp1002B_0998	D9QC41	Major facilitator superfamily domain protein
Cp1002B_1042	D9QBZ8	YdcZ inner membrane exporter
Cp1002B_1081	D9QBW2	DegV family protein
Cp1002B_1090	D9QBV3	Tryptophan-associated transmembrane protein
Cp1002B_1098	D9QBU6	TusA-like sulfuryltransferase, family YeeE/YedE
Cp1002B_1134	D9QBR0	VIT-1-like ferric transporter, family Ccc1
Cp1002B_1139	D9QBQ5	Glutamine-cyclotransferase
Cp1002B_1148	D9QBP5	YtxH-like protein
Cp1002B_1165	D9QBN0	GTP cyclohydrolase YbgI, interaction fator NIF3
Cp1002B_1166	D9QBM9	Zinc-Ribbon protein, nucleic acid ligand
Cp1002B_1182	D9QBL4	CsbD stress-response protein
Cp1002B_1210	D9QBJ0	Lipopolysaccharide assembly protein LapA
Cp1002B_1285	D9QBB4	TauE/SafE sulfide exporter
Cp1002B_1292	D9QBA6	Putative lumazine-binding protein
Cp1002B_1293	D9QBA5	Transmembrane lipid-transporter permease FtsX
Cp1002B_1350	D9QB49	Lipopolysaccharide assembly protein LapA
Cp1002B_1392	D9QB08	Putative DinB/YfiT-like metalloenzyme
Cp1002B_1414	A0A0M5KX29	UDP-glucose/GDP-mannose dehydrogenase
Cp1002B_1504	D9QAQ3	P-loop containing nucleoside triphosphate hydrolase
Cp1002B_1524	D9QAN3	Transcriptional regulator YbjN
Cp1002B_1549	A0A1L6D0H3	RNA-binding P-loop-containing hydrolase RapZ
Cp1002B_1597	D9QAF9	HTH-42 winged helix DNA-binding protein
Cp1002B_1611	A0A1L6D0C1	L-amino acid ligase C-terminal domain
Cp1002B_1661	D9QA99	Bifunctional nuclease—DNAse and RNAse
Cp1002B_1681	D9QA83	Phosphatidylglycerol lysyltransferase (mprF)
Cp1002B_1782	D9Q9Y9	HNH endonuclease
Cp1002B_1797	D9Q9X4	ACT-like enzyme
Cp1002B_1850	D9Q9S4	SWIM-like zinc finger protein
Cp1002B_1860	D9Q9R4	Cell wall-anchor domain LPxTG
Cp1002B_1865	D9Q9Q9	Ferric reductase FhuF
Cp1002B_1885	D9Q9N9	DivIVA-containing protein
Cp1002B_1903	D9Q9M1	Fosfomycin biosynthetic protein FomD
Cp1002B_1935	D9Q9I9	LGFP peptidoglycan-binding protein
Cp1002B_1942	D9Q9I1	Flavodoxin-like protein
Cp1002B_1944	D9Q9H9	PgaD-like protein
Cp1002B_1949	D9Q9H4	Bax1-inhibitor protein
Cp1002B_1975	D9Q9E9	Sulfolipid-1-addressing protein SfLAP
Cp1002B_1992	D9Q9D3	Substrate-binding periplasmic ABC lipoprotein
Cp1002B_2057	D9Q971	TcpE family protein
Cp1002B_2072	D9Q956	Cell wall synthesis potein CwsA
Cp1002B_2078	D9Q950	Actinobacterial holin-X protein, holin superfamily III

### Determination of virulence potential of target molecules

Among some auxiliary tools used, GIPSy software can generate genomic information related to the presence of virulence factors and the position of genes involved in pathogenicity mechanisms. For the *C. pseudotuberculosis* genome, we could identify 11 pathogenicity islands at all (Table S4). Our results show that all the total of 144 genes are found in these islands, which 65 are annotated as HP so far (~45%). From these HP, only 4 were functionally annotated in our study. All of them have their virulence potentials analyzed by the VirulentPred tool, to corroborate the results from GIPSy (Table 3), suggesting a possible role for these molecules in *C. pseudotuberculosis* pathogenicity.

**Table 3:** Pathogenicity islands present in *C. pseudotuberculosis* and the HP inserted on them, with their respective scores of virulence.

Pathogenicity Island	Uniprot ID (annotated function)	VirulentPred score	Final prediction
PAI 3	D9QEK1 (AMIN domain)	0.4889	Virulent
	D9QEL7 (restriction endonuclease HNHc)	0.6552	Virulent
PAI 5	D9QBU6 (TusA-like sulfuryltransferase family YeeE/YedE)	1.0606	Virulent
	D9QBV3 (Tryptophan-associated transmembrane protein)	1.0607	Virulent

### Identification of induced HP genes in abiotic stresses conditions

Since the transcriptomic data present in the work of Pinto et al. (2014) were generated in order to give a better understanding of the genes possibly involved in intracellular survival mechanisms, we integrated these information to analyze differentially expressed HP present in our dataset, where it was found 17 newly annotated targets induced upon abiotic conditions (Table S5), including products related to response to stress, formation of porins and cell wall synthesis, revealing that its products can play role in the modulation of the bacterial response against the host defense.

### PPI network

**Figure 3:**
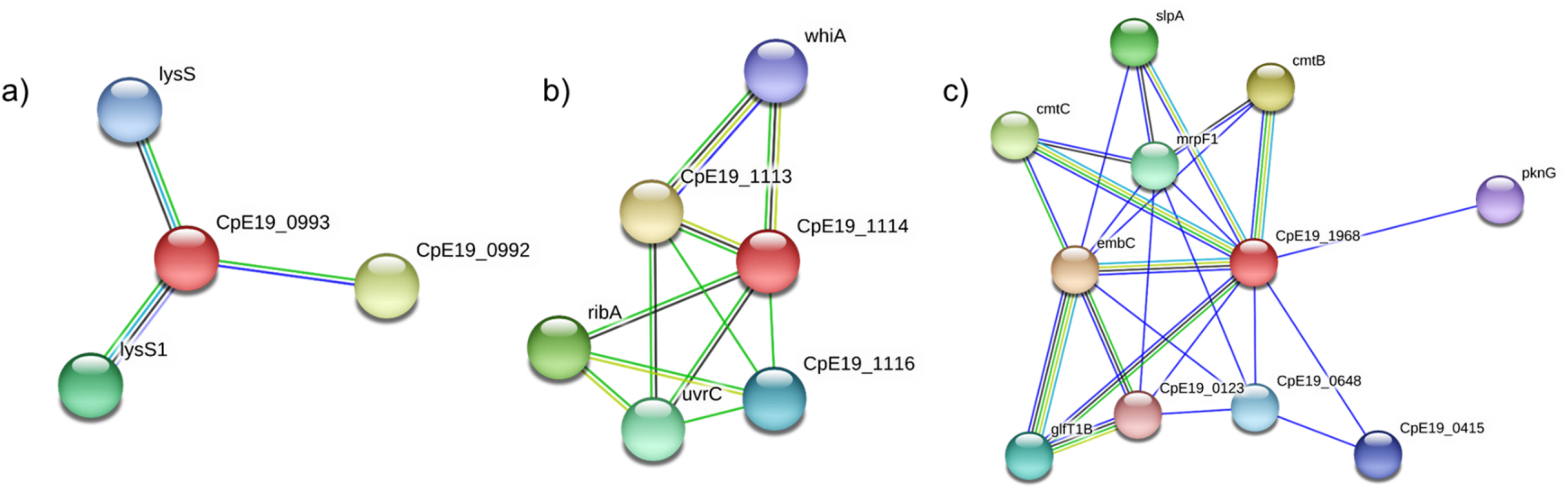
Protein–protein interaction networks generated by the STRING database. (A) D9QA83 (Phosphatidylglycerol transferase); (B) A0A1L6D0H3 (RNA-binding *P* loop-containing Hydrolase RapZ) and (C) D9QCX7 (Arabinan β-1,2 arabinofuranosyltransferase AftB).

Further, we highlighted the existence of associations between HP and other proteins, what can suggest some role of these molecules. Among our total set of 172 HP, 58 were discarded for not having high confidence interactions with other molecules. Regarding the remaining 114, 58 only possess interactions with unknown proteins. At last, 56 of our HP are related to known genes with high confidence. Thus, 104 of these genes were identified, classified with several functions, such as transcription factors, repair genes, DNA/RNA binding molecules and other functions. These genes can have important role in *C. pseudotuberculosis* biology, once they can allow response to intra and extracellular signals, as environmental and nutritional stimuli. For this reason, we selected 3 proteins to have its interactions analyzed individually. In Fig. 3, we exhibit protein–protein interaction networks of three selected targets—Phosphatidylglycerol lysyltransferase MprF, Hydrolase RapZ and Arabinan β-1,2-arabinofuranosyltransferase AftB, which are represented through connections, having their interaction intensities indicated by the thickness and color of lines. Target HP are illustrated by a homologous protein in *C. pseudotuberculosis* E19, indicated in red color.

### Three-dimensional structure of the three selected targets

From our set of molecules, we looked for relevant functions, due to their importance on the bacterial biology and pathogenicity mechanisms. Thus, the chosen proteins—Phosphatidylglycerol lysyltransferase MprF, Hydrolase RapZ and Arabinan β-1,2-arabinofuranosyltransferase AftB—were modelled using Phyre2 server.

In order to confirm the quality of the model and evaluate stereochemical features and possible mismatches in the molecular structure, it is important to consider the quality of protein structures. The 3Drefine was implemented to refine the selected models predicted by Phyre2 web-based homology modelling server. This method can improve global and local structure accuracy by two-step process involving optimization of hydrogen bonding network combined with atomic-level energy minimization on the optimized model using a composite physics and knowledge-based force fields (Bhattacharya et al., 2016).

**Figure 4:**
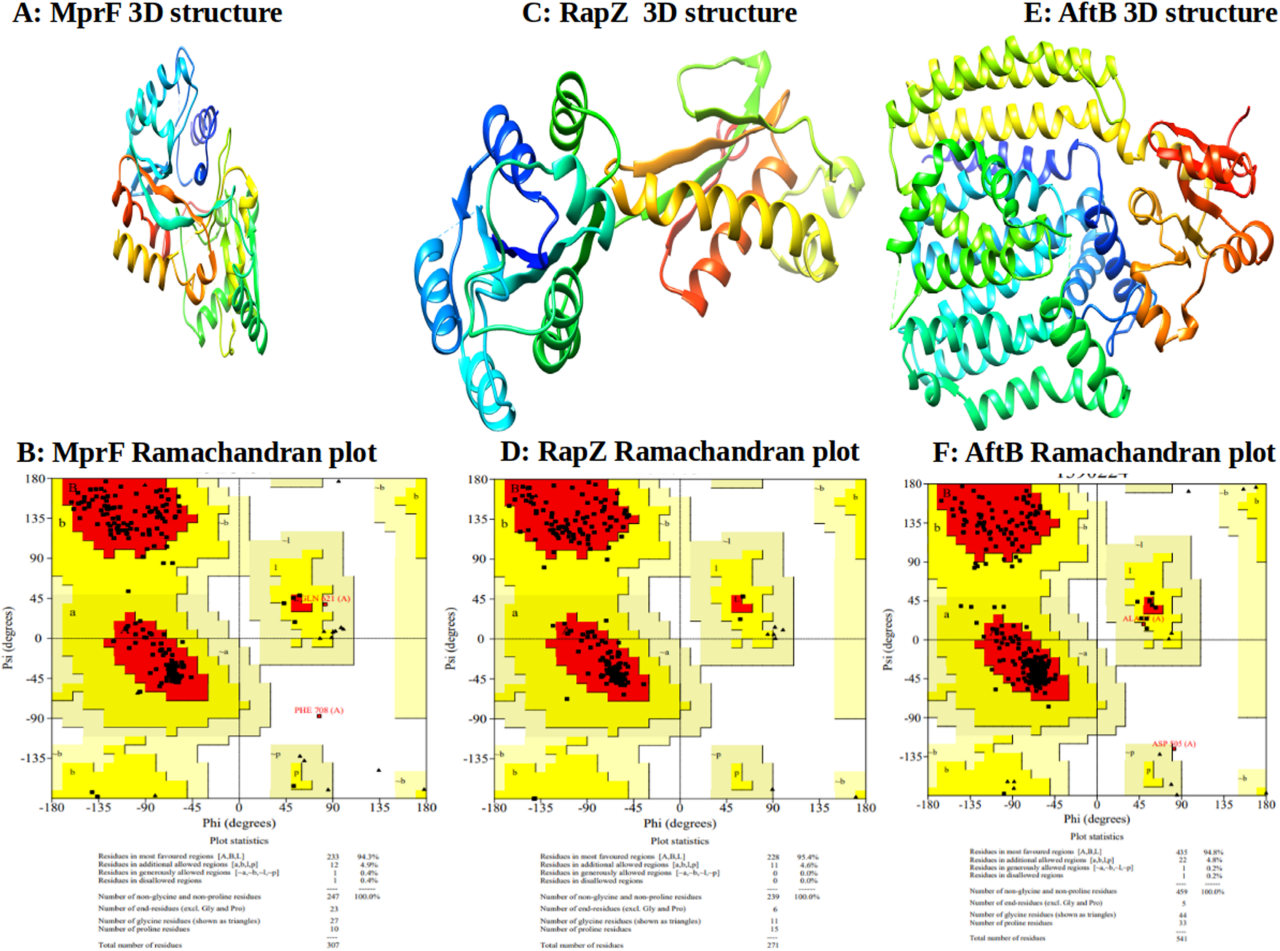
Structural models of the predicted proteins and its respectives Ramachadram plots (A) and (B) Phosphatidylglycerol transferase MprF; (C) and (D) Hydrolase RapZ and (E) and (F) Arabinan β-1,2 arabinofuranosyltransferase AftB.

The model corresponds to the lowest energy generated by the refinement. Furthermore, PROCHECK could generate the Ramachandran plot for the proteins Phosphatidylglycerol lysyltransferase MprF, Hydrolase RapZ and Arabinan β-1,2-arabinofuranosyltransferase AftB, what indicates that the generated models present excellent degree of reliability. The Phyre2 predicted the best tertiary structures of the proteins and the model with the highest confidence score were selected (Fig. 4).

Afterward, the 3D structures were refined through repeated structure perturbations and relaxation in the clusters side-chains, secondary structure elements, and loops of the models. The perturbations were done to improve the dihedral angles ψ and φ of the residues measured in the Ramachandran plot. From the refined structure models generated by the 3Drefine server, the models with the Ramachandram favored scores of MprF, RapZ and AftB presented 94.3%, 95.4% and 94.8% of AAs in favorable regions, respectively. The investigation of protein structure associated to the elucidation of its biological mechanisms and function is of vital importance. Thus, this result can significantly contribute to the characterization of this protein, previously known as a HP.

## Discussion

In recent years, several studies have focused on the annotation of proteins with unknown function, due to the importance of understanding the molecular mechanisms in different species, including pathogenic bacteria. The advance and availability of computational tools had a positive impact in the knowledge of the relationship between structure and function of proteins, also serving as a basis for the development of in vitro assays to the characterization of these proteins, which can reduce time and cost (Naqvi et al., 2016; Da Costa et al., 2018; Razali et al., 2019; Sen & Verma, 2020).

Generally, the pipelines of functional annotation based on sequence search are combined with the use of other approaches in order to optimize the reliability of the computational predictions. These additional steps include the analysis of gene products interactions, since proteins with related functions tend to have stronger interactions among them. Analysis of the three-dimensional structure and level of gene expression also can be very helpful in the discovery of proteins functions (Jun et al., 2017).

In our study, the combination of this methodology allowed the assignment of 80 newly annotated products in the core genome of *C. pseudotuberculosis*. These proteins can play role in different biological processes of this bacterium. Here, we pay special attention to some of them, based on the results of the supportive analysis that we have made.

Among the annotated proteins, we found a LGFP-containing protein (D9Q9I9), that has been described in several species of *Corynebacterium* and *Mycobacterium*. In *C. glutamicum* and *C. efficiens* this component was related to the PS1 protein, which can be anchored via LGFP tandem repeats when associated with cell wall (Adindla et al., 2004). The protein PS1 was described for its essential role in the transfer of mycolic acid residues to arabinogalactan and cell wall trehalose monocorinomicolate in *C. glutamicum*, a process of great importance for the maintenance of cellular permeability (Puech et al., 2000).

The integral membrane protein D9QBA5, found in members of the ABC transporter family is already been reported in *Escherichia coli*, interacting with a cytoplasmic ATPase (FtsE). The FtsEX complex can stimulate transport across the cell membrane (De Leeuw et al., 1999) and the role of this transporter complex in bacterial cell division has been reported in some microorganisms, such as *E. coli* and *M. tuberculosis* (Schmidt et al., 2004; Mir et al., 2006). A study with *Bacillus anthracis* revealed some specific bacterial genes which are important to facilitate antimicrobial activity against the pathogen, being one of them the *ftsX* gene. The results demonstrated that the functional alteration of *ftsX* produced vegetable bacilli with a chemokine-mediated resistance to death (Crawford et al., 2011). Thus, it is suggested that this protein can display a similar role in *C. pseudotuberculosis*.

The protein D9QD94 was predicted as the Multidrug Efflux Transporter AcrB, a component of the resistance nodulation cell division (RND), a group of transporters that are able to pump out several types of antibiotics of the cell through proton motive force (Murakami, 2008). Recently, a study demonstrated that AcrB has multiple potential substrate channels, using them according to them physical-chemical properties of the drugs, which allows the development of new antibiotics (Zwama et al., 2018).

The tryptophan-associated transmembrane protein, D9QBV3, belongs to the transcriptional unit operon *trp*, previously described in *E. coli*, which adds a group of genes necessary for the tryptophan biosynthesis (Yanofsky, 2007). Tryptophan is an amino acid used in protein synthesis and cell growth and is regarded as a biosynthetic precursor of a large number of natural microbial products. Thus, it is of special interest in the discovery and development of drugs (Alkhalaf & Ryan, 2015). Several studies with *C. glutamicum* have already expressed the importance of tryptophan biosynthesis for metabolic engineering (Yu et al., 2015; Sun et al., 2016).

Another identified protein was porin PorA (D9QE95), which exhibited relation to the main permeability pathway of hydrophylic solutes among *Corynebacterium* species. PorA is a channel-forming protein found in the bacterial cell wall, which presents two main characteristics: it forms large pores and it possesses positive charge, resulting in selectivity for cations (Soltan Mohammadi et al., 2013).

Since microorganisms are prone to different types of abiotic stresses (such as temperature and pH alteration, presence of toxic molecules, nutrient availability, etc.), we decided to analyze the gene expression level of the predicted HP, based on data previously available.

The ability of adapting to osmotic changes in the cellular microenvironment can be crucial to bacterial survival and links directly to virulence in pathogenic bacteria, once increases or decreases in osmolarity can lead to cell death by plasmolysis or cytolysis. It is known that pathogenic bacteria have developed sophisticated signal transduction systems to control the expression of virulence determinants in response to environmental stresses, and changes in osmolarity can contribute to the expression of these genes (Sleator & Hill, 2001).

Also, pH alteration can negatively affect the metabolism, causing damage to macromolecules. For some pathogens, resistance to acid environment is fundamental to colonize the environment of the host. It is reported that under acid stress conditions, the induction of genes involved in the processes of oxidoreduction and cellular adhesion were of great importance, because they are strongly related to virulence (Pinto et al., 2014).

Furthermore, some genes related to thermal shock, cold shock and virulence can have its expressions affected by temperature alterations (Narberhaus, Waldminghaus & Chowdhury, 2006). The increase in temperature will directly affect the level of expression of these genes. It is reported that this alteration of heat-shock regulation can influence virulence potential because the bacterium loses the ability to establish a chronic infection (Gomide et al., 2018).

Regarding the annotated products analyzed by its interactions networks, the phosphatidylglycerol lysyltransferase (D9QA83) was previously described in *Staphylococcus aureus* and is involved in bacterial virulence from resistance process to cationic antimicrobial molecules (CAM) produced by the host immune system (defensins, catatelicidins), or by competing microorganisms (bacteriocins) (Staubitz et al., 2004). MprF is a membrane protein that acts on bacterial resistance from the production of lysylphosphatidylglycerol (L-PG). L-PG is formed when MprF assigns a positively charged lysyl group to membrane bound anionic phosphatidylglycerol, which causes that membrane to become positively charged, thereby decreasing bacterial affinity by CAMs (Khatib et al., 2016). Some studies have already described the essential role of this protein in the mechanism of resistance to daptomycin, a last-line defense antibiotic for the treatment of infections caused by Gram-positive bacteria (Yang et al., 2018). Through protein interaction network, we found that this molecule may be related to the constitutive gene *lysS*, that encodes for Lysine-tRNA ligase, responsible for the attachment of lysine to its cognate tRNA during the synthesis of proteins, which is expressed under all normal growth conditions in *E. coli*, being responsible for the normal tRNA^Lys^ charging activity (Onesti, Miller & Brick, 1995).

The adapter protein Hydrolase RapZ (A0A1L6D0H3) guides the biochemical reactions to provide precursors for the assembly of the cell wall and bacterial outer membrane (Gonzalez et al., 2017). Thus, it is possible to assume that this protein, as an important metabolic component, must also play a fundamental role in the microorganism under study. The in silico predictions revealed that this protein may be related to the gene *uvrC*, member of the *uvrABCD* complex involved in the DNA repair mechanism (Prammananan et al., 2012). Also, another gene related to RapZ is *ribA*, involved in the biosynthesis of riboflavin, which is itself part of cofactor biosynthesis.

Arabinogalactan is a structural polysaccharide present in the cell wall of the CMNR members serving to connect peptidoglycan with the outer mycolic acid layer, forming the complex mycolyl-arabinogalactan-peptidoglycan (mAGP). In this study we are able to assign function to the protein AftB (D9QCX7), an arabinofuranosyl transferase that is, involved in the arabinogalactan biosynthesis process (Seidel et al., 2007; Jankute et al., 2017).

Jankute et al. (2014) elucidated the interaction network in vivo of proteins related to the *C. glutamicum* cell wall synthesis, revealing that the cluster *aftABC* and *emb* strongly interact, because they constitute an inner membrane complex involved in the assembly of the arabinan domain. Enzymes related to this process represent a potential new drug target, since the complex mAGP play pivotal role in intracellular survival and virulence in *C. pseudotuberculosis* and other related-pathogens (Mdluli & Spigelman, 2006; Burkovski, 2013). Altogether, these studies support our in silico prediction and have shed further light on the complexities of Corynebacteriaceae cell wall biosynthesis.

## Conclusions

The present study was able to elucidate functional characteristics of HP shared by several strains of *C. pseudotuberculosis* biovar *ovis*, exhibiting important biological features of this subgroup that were not yet described. Considering the harmful effects that CLA can cause in the veterinary and economic fields globally, this research provided new information about proteins with unknown functions so far that are present in the set of shared genes in several strains this pathogen. Hence, it was possible to assign physicochemical definitions, subcellular localization, gene expression levels and dynamics of interaction among other proteins. Besides that, other evidences as the presence of HP in pathogenicity islands suggest the virulence potential of some of the determined sequences. At last, we constructed the structural model of three annotated HP with high-confidence. Together, our findings point to possibilities for studies involving these targets, specially to investigate the role of these products in vitro, as well as their potential to be involved in pathogenesis and its use for the development of new therapeutic targets against CLA.

## Supplemental Information

10.7717/peerj.9643/supp-1 Supplemental Information 1 Pan-genome profile of 32 *C. pseudotuberculosis* biovar *ovis* strains.

10.7717/peerj.9643/supp-2 Supplemental Information 2 Theoretical prediction of physicochemical parameters, subcellular localization and protein function for 172 HP.

10.7717/peerj.9643/supp-3 Supplemental Information 3 List of GO terms provided by the GO feat platform.

10.7717/peerj.9643/supp-4 Supplemental Information 4 Prediction of pathogenicity islands in *C. pseudotuberculosis* *ovis*.

10.7717/peerj.9643/supp-5 Supplemental Information 5 Fold change values of the newly anotated genes differentially expressed upon abiotic stress conditions.

## Additional Information and Declarations

### Data Availability

The following information was supplied regarding data availability: This work did not generate raw data.
